# Adsorption of Proteins on m-CPPD and Urate Crystals Inhibits Crystal-Induced Cell Responses: Study on Albumin-Crystal Interaction

**DOI:** 10.3390/jfb10020018

**Published:** 2019-04-25

**Authors:** Felix Renaudin, Stéphanie Sarda, Laure Campillo-Gimenez, Childérick Séverac, Thibaut Léger, Cédric Charvillat, Christian Rey, Frédéric Lioté, Jean-Michel Camadro, Hang-Korng Ea, Christèle Combes

**Affiliations:** 1 Université Paris 7 Denis Diderot, Inserm UMR 1132 Bioscar, Hôpital Lariboisière, Centre Viggo Petersen, 75010 Paris, France; felix.renaudin@inserm.fr (F.R.); campillo.laure@gmail.com (L.C.-G.); frederic.liote@aphp.fr (F.L.); 2 CIRIMAT, Université de Toulouse, CNRS, Université Toulouse 3, Toulouse INP-ENSIACET, 31030 Toulouse, France; stephanie.sarda@iut-tlse3.fr (S.S.); cedric.charvillat@ensiacet.fr (C.C.); Christian.rey@ensiacet.fr (C.R.); 3 ITAV-CNRS, Université de Toulouse, CNRS, 31106 Toulouse, France; childerick.severac@cnrs.fr; 4 Institut Jacques Monod, UMR7592 CNRS, Université Paris Diderot, 75013 Paris, France; thibaut.leger@ijm.fr (T.L.); jean-michel.camadro@ijm.fr (J.-M.C.)

**Keywords:** chondrocalcinosis, gout, Interleukin-1, proteins, m-CPPD crystals, albumin, adsorption

## Abstract

The biological effects and cellular activations triggered by monosodium urate (MSU) and calcium pyrophosphate dihydrate (monoclinic: m-CPPD) crystals might be modulated by protein coating on the crystal surface. This study is aimed at: (i) Identifying proteins adsorbed on m-CPPD crystals, and the underlying mechanisms of protein adsorption, and (ii) to understand how protein coating did modulate the inflammatory properties of m-CPPD crystals. The effects of protein coating were assessed in vitro using primary macrophages and THP1 monocytes. Physico-chemical studies on the adsorption of bovine serum albumin (BSA) upon m-CPPD crystals were performed. Adsorption of serum proteins, and BSA on MSU, as well as upon m-CPPD crystals, inhibited their capacity to induce interleukin-1-β secretions, along with a decreased ATP secretion, and a disturbance of mitochondrial membrane depolarization, suggesting an alteration of NLRP3 inflammasome activation. Proteomic analysis identified numerous m-CPPD-associated proteins including hemoglobin, complement, albumin, apolipoproteins and coagulation factors. BSA adsorption on m-CPPD crystals followed a Langmuir-Freundlich isotherm, suggesting that it could modulate m-CPPD crystal-induced cell responses through crystal/cell-membrane interaction. BSA is adsorbed on m-CPPD crystals with weak interactions, confirmed by the preliminary AFM study, but strong interactions of BSA molecules with each other occurred favoring crystal agglomeration, which might contribute to a decrease in the inflammatory properties of m-CPPD crystals. These findings give new insights into the pathogenesis of crystal-related rheumatic diseases and subsequently may open the way for new therapeutic approaches.

## 1. Introduction

Calcium orthophosphates such as carbonated-apatites, calcium pyrophosphate dihydrate (CPPD) and monosodium urate (MSU) are three main types of crystals associated with rheumatic diseases, responsible for hydroxyapatite rheumatism, CPP deposition disease and gout, respectively.

Prevalence of these crystal depositions is high, occurring for example in more than 17.5% of people over 75 years for CPPD crystals, and between 0.9 and more than 10% for MSU crystals [1,2,3]. Monoclinic and triclinic CPPD phases (m-CPPD and t-CPPD respectively) are the two CPPD crystals identified in human tissues, while the existence of precursor forms such as monoclinic calcium pyrophosphate tetrahydrate beta (m-CPPTβ), and amorphous calcium pyrophosphate (a-CPP) phases have been suggested by in vitro studies [4,5,6]. Although these crystals remain mostly asymptomatic, they can induce recurrent, self-resolving and interleukin (IL)-1β-driven inflammation reactions [7,8,9]. How these crystals can switch from an asymptomatic state to an inflammatory one is still unknown. Similarly, how the crystal-induced inflammation self-resolves remains not completely understood. We speculated that modification of protein coating on the crystal surface might modulate immune cell responses and contribute to these different clinical phenotypes.

Studies show that apatite, MSU and CPPD crystals harbor different inflammatory potentials, which can rely on their physico-chemical characteristics, including chemical composition, crystal shape, size and surface properties [10,11,12,13,14,15,16,17,18]. Recently, we confirm that among CPPD crystals, m-CPPD crystals are the most inflammatory ones, while the amorphous phases do not have inflammatory property [14,17,19]. The inflammatory potential of CPPD crystals depends on their capacity to activate MAPK pathways and the nuclear factor Kappa B (NF-κB) [17]. Lebre et al. observe that the inflammatory potential of hydroxyapatite particles varies with their sizes and shapes [10]. Thus, small needle-shaped particles induce a higher IL-1β secretion and neutrophils recruitment than smooth, spherical particles of comparable size, or than larger particles [10]. These results suggest that the clinical features might be secondary to the presence of different types of crystals which might modify during time. Thus, Swan et al. observe that the ratio of m-CPPD/t-CPPD crystals is higher in inflammatory synovial fluids than in non-inflammatory synovial fluids [20]. We hypothesized that CPPD crystal phases exhibited different protein adsorption properties which might explain their capacity to activate immune cells.

Thus, the inflammatory potential of different crystals also depends on their surface properties that can modulate crystal/cell interactions either through their surface charge and/or through proteins coated on their surface. MSU crystal surfaces can be coated by several proteins, including albumin, fibrinogen, fibronectin, lysozyme, bovine serum albumin (BSA), ovalbumin, immunoglobulin (Ig), apolipoproteins (Apo) and high and low density lipoproteins (HDL and LDL) [21,22,23,24,25]. MSU crystal inflammatory properties vary according to proteins adsorbed on their surface. Thus, adsorption of ApoA, ApoE, human serum or HDL inhibits MSU crystal-induced inflammation, while IgG coating enhances it [23,24,25,26,27,28,29]. Moreover, proteins coated on MSU crystals change during inflammation phases, and might contribute to the self-limiting nature of gout flare [27]. Similarly, nanoparticles (NP) are rapidly coated with proteins, resulting in the formation of the so-called protein corona. The adsorbed proteins modulate NP interactions with the innate immune system, and depending on coated-proteins innate immune cell responses can have opposing effects, with either a suppression or a stimulation effect [30,31]. Contrary to MSU crystals and NP, the effect of protein coating on CPPD crystal-induced inflammation, described 30 to 40 years ago, is scarcely investigated, and using mostly t-CPPD crystals, which are less inflammatory than m-CPPD crystals [13,17,21,22,32,33]. Moreover, none of these studies have assessed the effects of protein coating on IL-1β production, which is the main inflammatory cytokine involved in crystal-induced inflammation. We aimed to assess the effects of protein coating on CPPD crystal-induced inflammation, to identify proteins adsorbed on CPPD crystals using proteomic technology, and to study the mechanisms involved in these interactions.

Indeed, how proteins adsorb onto crystal surfaces remains unclear. Protein adsorption could be related to either a charge-mediated process or a surface-ion-exchange process, as described with calcium orthophosphate crystals [34,35,36]. Both MSU and CPPD crystals have a negative zeta potential, and could interact with positively charged proteins. However, this hypothesis needs to be evaluated, and the mechanism of protein adsorption onto CPPD crystals thoroughly studied.

## 2. Results

### 2.1. Adsorption of Serum Proteins Inhibits Crystal-Induced Inflammation

**Figure 1:**
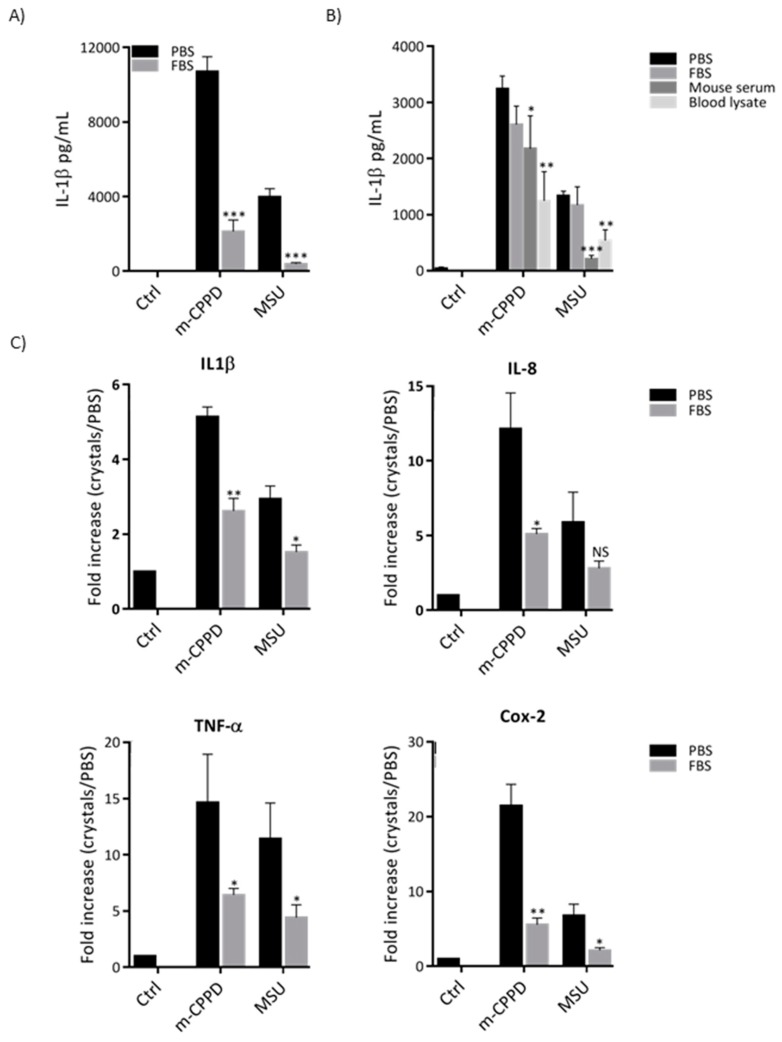
Adsorption of serum proteins inhibits crystal-induced inflammation. THP-1 or mouse BMDMs were primed the day before the experiment. After 6 h of crystal stimulation, supernatants and total RNA were collected. (**A**) Human IL-1β concentrations from THP-1 cultures (n = 6) were quantified by ELISA. (**B**) Mouse IL-1β concentrations from BMDM cultures (n = 6) were quantified by ELISA. (**C**) IL-1β, IL-8, TNF-α and Cox-2 gene expression from THP-1 cells were quantified by RT-qPCR (n = 4). Multiple t-test with FDR correction between uncoated and FBS-coated crystals were performed: * *p* < 0.05; ** *p* < 0.01; *** *p* < 0.001.

We first assessed the effect of MSU and m-CPPD crystals which were pre-incubated with fetal bovine serum (FBS) on IL-1β secretion by the human cell line THP1. We observed that both FBS-coated MSU and m-CPPD crystals were less inflammatory than naked MSU and m-CPPD crystals (Figure 1). Thus, FBS coating decreased by more than 75% and 90% IL-1β production induced by naked m-CPPD and MSU crystals, respectively. Interestingly, FBS coating did not modulate MSU and m-CPPD crystal-induced IL-1β production when using mouse bone marrow derived macrophages (BMDM) suggesting specie specificity. Indeed, when we pre-incubated MSU and m-CPPD crystals with mouse serum or mouse blood lysate, IL-1β production was dramatically decreased compared to naked crystals (Figure 1B). Next, we wondered whether FBS coating could modulate the effects of MSU and m-CPPD crystals on other inflammatory cytokines in THP1. Our results showed that pre-incubation of m-CPPD and MSU crystals in FBS inhibited their capacity to induce the expression of IL-1β, TNF-α, IL-8 and cyclooxygenase (Cox)-2 genes (Figure 1C).

### 2.2. Proteomic Analysis of Proteins Adsorbed on m-CPPD Crystals

To determine which of the FBS proteins are associated with the inhibition of the inflammatory response induced by m-CPPD crystals we performed proteomic analysis of the proteins adsorbed on m-CPPD crystals after 30 min of incubation in FBS at 37 °C. Proteomic analysis identified 30 proteins with a mascot score higher than 40 (Table 1). Among these proteins 10 had a mascot score higher than 100 and were identified as fetal hemoglobin subunit alpha and beta, fibrinogen, alpha-trypsin inhibitor heavy chain H4, alpha-2-HS glycoprotein precursor, albumin, complement C4 precursor and Apo A-1 and A-2 precursors. Interestingly, most of these proteins had been previously identified on MSU crystals [23,27,29] except for albumin which had little affinity to bind MSU crystals [22].

### 2.3. Albumin Coating Inhibits m-CPPD Induced Inflammation

**Figure 2:**
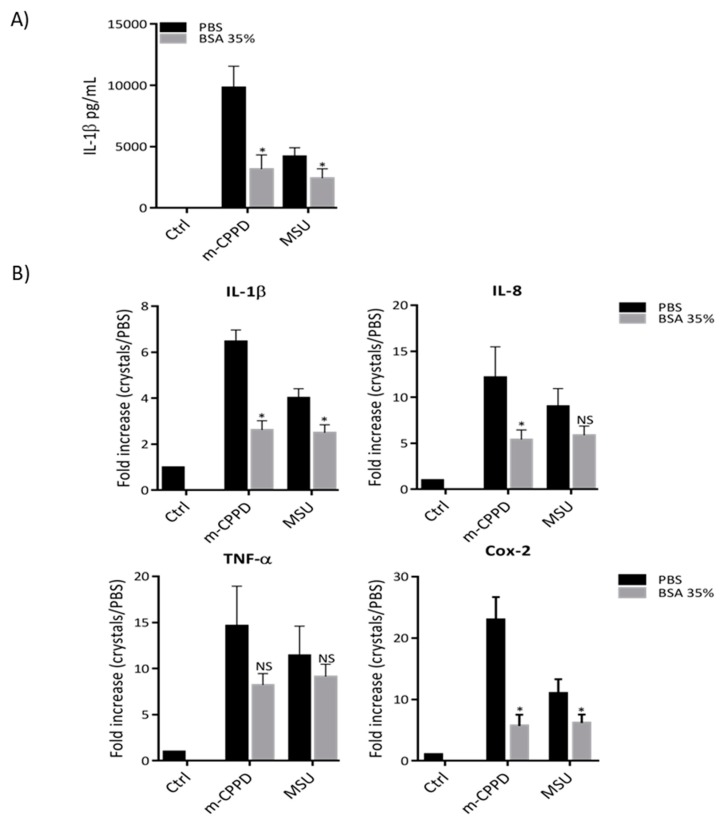
BSA-coated crystals inhibit crystal induced inflammation. THP-1 cells were primed the day before experiment. After 6 h of crystal stimulation, supernatants and total RNA were collected. (**A**) Human IL-1β concentrations were quantified by ELISA in the supernatants (n = 3). (**B**) mRNA quantification of IL-1β, IL-8, TNF-α and Cox-2 gene expression was performed by RT-qPCR (n = 3). Multiple t-test with FDR correction between uncoated and crystals pre-incubated with BSA 35% (*): * *p* < 0.05; ** *p* < 0.01; *** *p* < 0.001.

As albumin is the most abundant protein in serum and was identified with high mascot score in the proteomic analysis, we hypothesized that BSA coating on MSU and m-CPPD crystals contributed to the reduction of inflammation after FBS pre-incubation. To address this question, we stimulated THP1 cells with MSU and m-CPPD crystals which were pre-incubated with BSA. Then, we observed that actually pre-incubation of m-CPPD and MSU crystals with BSA 35% inhibited their capacity to induce IL-1β production (Figure 2). Similarly, the induction of IL-1β, IL-8 and Cox-2 gene expression by BSA-coated MSU and m-CPPD crystals was less important than the induction generated by naked MSU and m-CPPD crystals (Figure 2B). Interestingly, BSA coating did not modulate the effects of MSU and m-CPPD crystals on TNF-α gene expression suggesting that protein coating on crystal surface altered specific intracellular pathways.

### 2.4. Protein Adsorption Inhibits Crystal-Induced Inflammation through Inhibition of Membrane-Crystal Interaction

**Figure 3:**
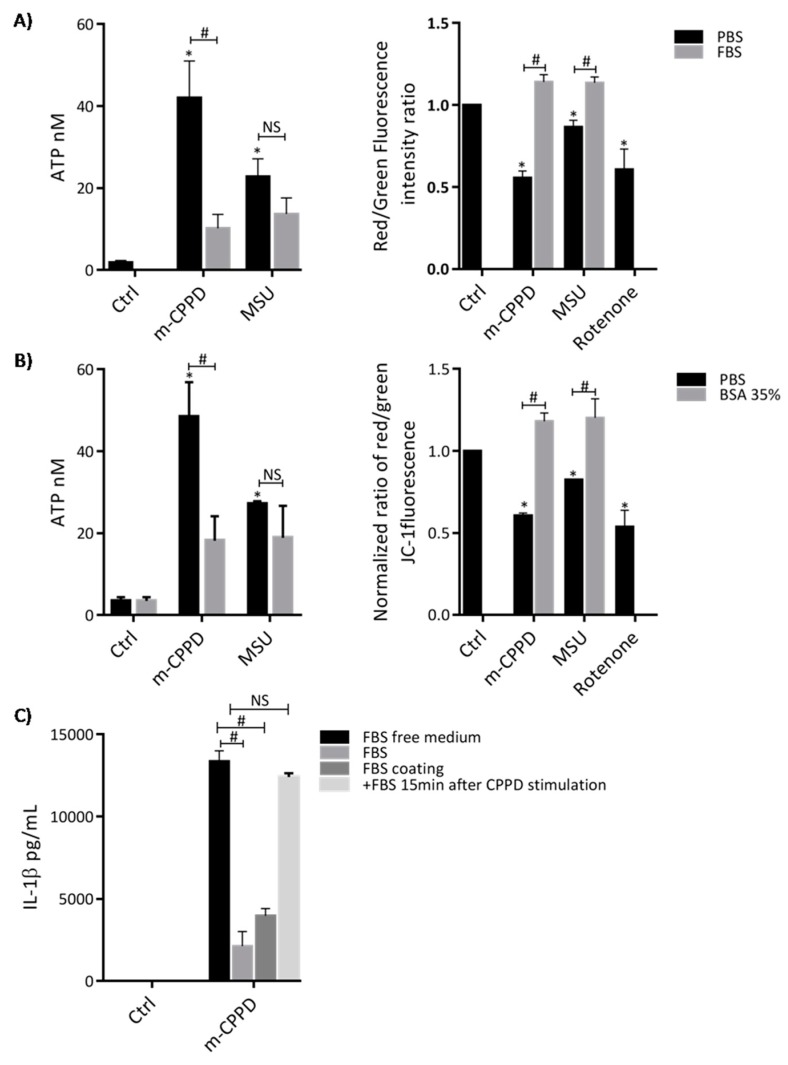
Adsorption of serum proteins on m-CPPD crystals modulates extracellular ATP production and mitochondrial membrane potential. THP-1 cells were primed the day before the experiment. (**A**) After 2 h of crystal stimulation, supernatants were collected and ATP concentrations were quantified (n = 3). (**B**) After 30 min of crystal stimulation, cells were incubated with the JC-1 probe and then analyzed by flow cytometry (n = 3). The decrease of red/green fluorescence intensity ratio indicated mitochondrial transmembrane depolarization. Kruskal-Wallis test with FDR correction compare to Ctrl (*) or uncoated crystals (#): * *p* < 0.05; # *p* < 0.05. (**C**) Cells were stimulated with (i) uncoated m-CPPD crystals in either serum-free medium or serum-containing medium; (ii) with FBS-coated m-CPPD crystals in serum-free medium or iii) with uncoated m-CPPD crystals during 15 min in serum-free medium and then FBS was added in the culture medium. Supernatants were collected after 6 h of stimulation and human IL-1β (n = 3) concentrations were quantified by ELISA. Multiple t-test with FDR correction between uncoated and FBS coated crystals (*).

Then, we investigated how did protein adsorption suppress m-CPPD-induced inflammation, especially how did BSA-coating inhibit m-CPPD induced IL-1β production. IL-1β production is a two-step process encompassing the production of pro-IL-1β through NF-κB activation and the maturation of pro-IL-1β through NLRP3 inflammasome activation and caspase-1 cleavage [8,17]. We first observed that m-CPPD and MSU crystals stimulated strong increase of ATP secretion by THP1 cells and depolarization of mitochondrial membrane potential, two well-described mechanisms of NLRP3 inflammasome activation (Figure 3A) [37,38]. Mitochondrial transmembrane depolarization was evidenced by the decrease of JC-1 red/green fluorescence intensity ratio under MSU and m-CPPD crystal stimulation. JC-1 (5,5’,6,-’, tetrachloro-1,1’,3,3’ tetraethylbenzimi-dazolylcarbocyanide iodide) dye formed in mitochondrial with high transmembrane potential j-aggregate complexes that exhibited high red fluorescence while in mitochondrial with low transmembrane potential it remained in the monomeric forms with green fluorescence. Consequently, mitochondrial depolarization was indicated by a decrease in red/green fluorescence intensity ratio [39]. In this experiment, we used rotenone, an inhibitor of mitochondrial electron transport chain complex I, as positive control to induce mitochondrial transmembrane depolarization. Figure 3A showed that m-CPPD crystals decreased JC-1 red/green fluorescence ratio at the same level than rotenone treatment. Next, we showed that both FBS and BSA pre-incubation significantly inhibited m-CPPD crystal-induced ATP production and depolarization of mitochondrial membrane potential (Figure 3A,B). Interestingly FBS or BSA coating did not alter MSU crystal-induced ATP production while it did abrogate MSU crystal-induced depolarization of mitochondrial membrane potential (Figure 3A,B). These results suggested that m-CPPD and MSU crystals induced cell activation through different signaling pathways. As MSU crystal-activated NLRP3 inflammasome involves crystal/membrane interactions, we assessed whether the inhibitory effect of FBS coating on m-CPPD crystals was secondary to these interactions [40,41,42]. To address this question, we let uncoated m-CPPD crystals interact with cells during 15 min and then added in the culture medium 10% of FBS. By doing this, we observed that adding FBS in the culture medium after cell stimulation with uncoated m-CPPD crystals did not modify the amount of IL-1β production induced by naked m-CPPD crystals (Figure 3C). In contrary, the amount of IL-1β was very low if cells were stimulated with uncoated m-CPPD crystals in medium containing FBS or with FBS-coated m-CPPD crystals (Figure 3C). Altogether these results suggested that adsorption of serum proteins on m-CPPD crystals modulates their cellular effects through disturbances of crystal-membrane interactions.

### 2.5. Physico-Chemical Study of the Adsorption of BSA on m-CPPD Crystals and Its Model of Adsorption

**Figure 4:**
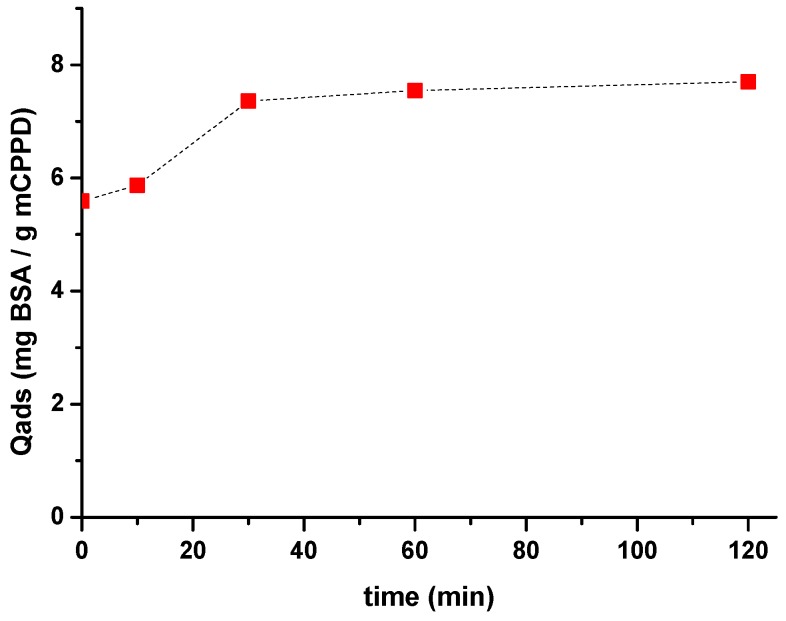
Kinetic of BSA adsorption on m-CPPD crystals at 37 °C and pH 7.4. The initial BSA concentration was fixed to 0.5 g/L.

To understand the mechanism of adsorption of protein on m-CPPD crystals, we studied the adsorption of BSA as a protein model on m-CPPD synthetic crystals in aqueous medium (NaCl 0.15 M) with different concentrations of BSA at 37 °C and pH 7.4. In a first approach, the adsorption kinetic of BSA on m-CPPD crystals was plotted by following the evolution of BSA quantity adsorbed (Q_ads_) as a function of time (Figure 4) with initial concentration of BSA fixed to 0.5 g/L. The results show that Q_ads_ increased quickly with time and a plateau was reached within the first 30 min. The contact time for the adsorption experiments was then fixed to 30 min to limit the evolution and potential dissolution of m-CPPD crystals during the adsorption test.

**Figure 5:**
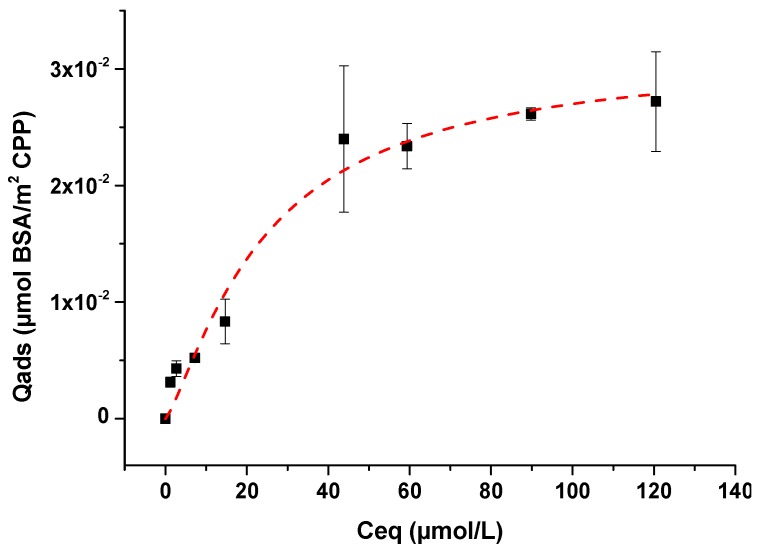
Isotherm of BSA adsorption on m-CPPD crystals (n = 3) at 37 °C and pH 7.4. The dashed red curve indicates curve-fitting with the Langmuir-Freundlich model (r^2^ = 0.97).

In a second step, the adsorption experiments of BSA on m-CPPD crystals were performed using the selected contact time with initial BSA concentration varying from 0 to 10 g/L. The remained concentration of BSA in the supernatant after adsorption was titrated by UV spectroscopy. Figure 5 showed the adsorption isotherm obtained by plotting the evolution of the amount of BSA adsorbed onto m-CPPD from dilute solutions (Q_ads_ in μmol·m^−2^) as a function of its remaining equilibrium concentration in solution (C_eq_ in mmol·L^−1^). Small quantity of albumin adsorbed onto m-CPPD crystals was observed compared with the adsorption data of proteins or others biomolecules on inorganic crystal surfaces like calcium phosphates [34,35,36]. These results showed that BSA/m-CPPD crystals interactions remained quite low.

To understand the adsorption behavior, mathematical analysis was applied to model the adsorption isotherm of BSA onto m-CPPD crystals. Although fitting to the Langmuir isotherm model leads to an acceptable r^2^ coefficient (0.96), the isotherm did not reach a plateau and the quantity of BSA molecules adsorbed was low. While the model of Freundlich leads to poor correspondence (r^2^ = 0.93), the Langmuir-Freundlich model best described the present data (r^2^ = 0.97) as seen in Figure 5 [43]. This model is generally encountered when the adsorbed molecules present low affinity for mineral surfaces (low interaction adsorbate/surface and low quantity adsorbed). The adsorption parameters N, K and n can be calculated from the Langmuir-Freundlich equation (also known as Sip’s equation), Equation (1):

(1) $$Q_{ads}=N\times \frac{{\left(K\times C_{eq}\right)}^{n}}{1+{\left(K\times C_{eq}\right)}^{n}}$$

where *N* is the maximum adsorption coverage (µmol·m^−2^), *K* the affinity constant of adsorption (L·mmol^−1^) and n the constant of heterogeneity. Applying this model, the values obtained for the adsorption parameters of BSA/m-CPPD isotherm are: *N* = 0.031 ± 0.005 µmol·m^−2^, *K* = 0.04 ± 0.02 L·mmol^−1^ and *n* = 1.4 ± 0.3. The parameters of adsorption *N* and *K* determined were very low compared with adsorption parameters of biomolecules or drugs adsorbed on apatitic calcium phosphates for example [34,35,36]. These values reflected the small quantity of adsorbed BSA and the low affinity of BSA for m-CPPD surface. The small amount adsorbed could also be related to the size (steric dimension) and conformation of BSA macromolecules on crystal surface. Moreover, n parameter value appeared greater than 1 suggesting the existence of a high affinity of the adsorbed molecules with each other. Often molecules could form multilayers onto mineral surface, i.e., some are adsorbed on already adsorbed molecules, or at least could induce agglomeration of adsorbed particles of CPPD. This behavior has been recently described for the adsorption of some drug molecules onto calcium phosphate compounds also described by Sips model [36,44].

Analysis of phosphorus and calcium concentrations in solution after adsorption experiment using ICP OES spectrometer were plotted as a function of the quantity of BSA adsorbed (Figure A3). The Ca and P amounts were not affected by BSA adsorption: they remained low and stable during the adsorption, mainly related to m-CPPD dissolution equilibrium. Ions in solution do not participate to the adsorption process contrary to Langmuir adsorption of biomolecules observed onto mineral surface like calcium phosphates [45]. The mechanism of adsorption of BSA on m-CPPD crystals cannot be described by an ion exchange process between BSA and ionic species on the surface of m-CPPD crystals during adsorption. In accordance with the Sips model proposed, the adsorption of BSA onto m-CPPD crystals is related to a weak interaction protein/crystals.

**Figure 6:**
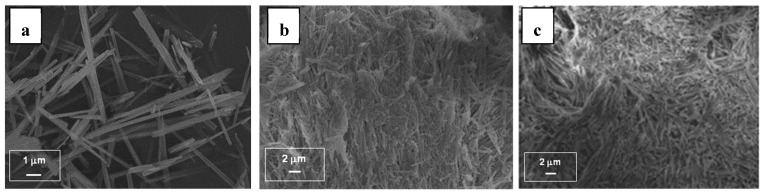
SEM micrographs of the synthesized m-CPPD crystals (**a**) before adsorption experiment, (**b**) after BSA adsorption experiment and (**c**) after in vitro cell test.

**Figure A1:**
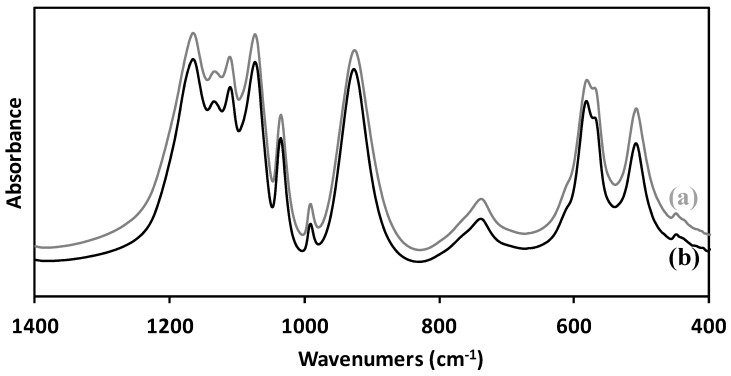
Fourier-transform infrared spectroscopy (FTIR) spectrum of m-CPPD synthetic powder (**a**) before bovine serum albumin (BSA) adsorption; (**b**) after BSA adsorption, at 37 °C, and pH 7.4 (with initial [BSA] in solution, fixed to 2 g/L). The spectra were recorded over a wavelength range of 400–1400 cm^−1^.

**Figure A2:**
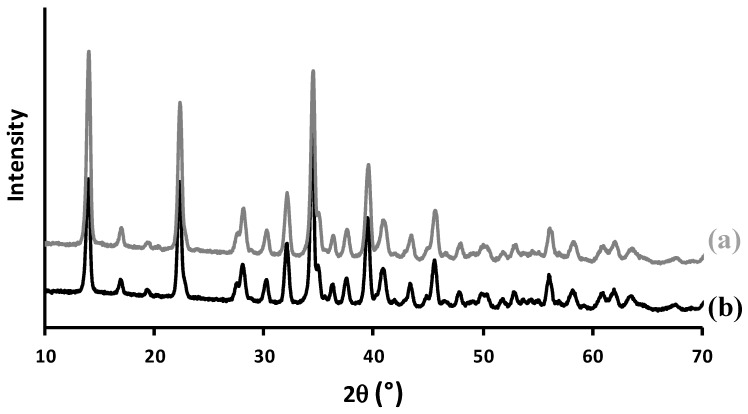
X-ray diffraction (XRD) diagram of m-CPPD synthetic powder (**a**) before BSA adsorption; (**b**) after BSA adsorption at 37 °C and pH 7.4 (with initial [BSA] in solution fixed to 2 g/L).

**Figure A3:**
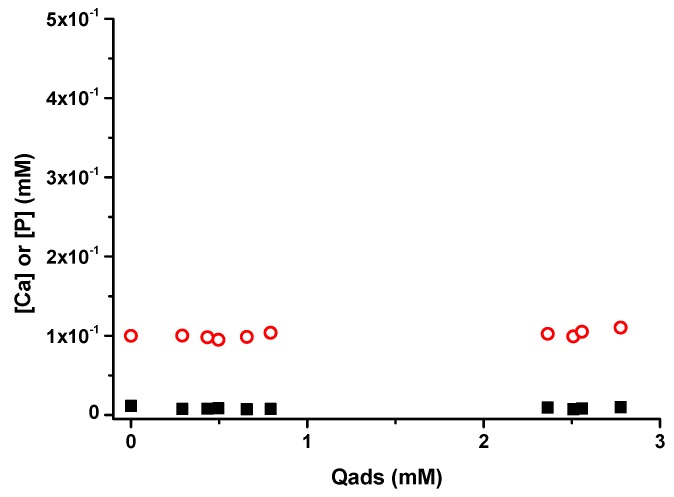
Concentration of Ca (open red circles o) and P (solid black squares ■) content released (mM) in solution as a function of BSA quantity (Qads) adsorbed (mM) on m-CPPD crystals (n = 3). Very low amounts of P and Ca were released in solution after adsorption: measured concentrations were close to the detection limit of the inductively coupled plasma-atomic emission spectroscopy (ICP-OES) spectrometer.

To better understand the mechanism of adsorption, we also characterized the solid after adsorption experiments. FTIR spectroscopy and XRD analyzes (Figure A1 and Figure A2) showed that no other crystalline phase or change in crystallinity or detection of the presence of any other groups occurred. No change in crystal size and morphology of m-CPPD crystals was observed by SEM after adsorption of BSA (Figure 6a,b). However the SEM images evidenced a high agglomeration of the crystals after BSA adsorption. The observation of such crystals agglomeration was in accordance with the Sips model of adsorption proposed and possible interactions between BSA molecules. This agglomeration of m-CPPD crystals were observed after in vitro cell tests (Figure 6c), and could be responsible for the decrease of the inflammatory response.

**Figure 7:**
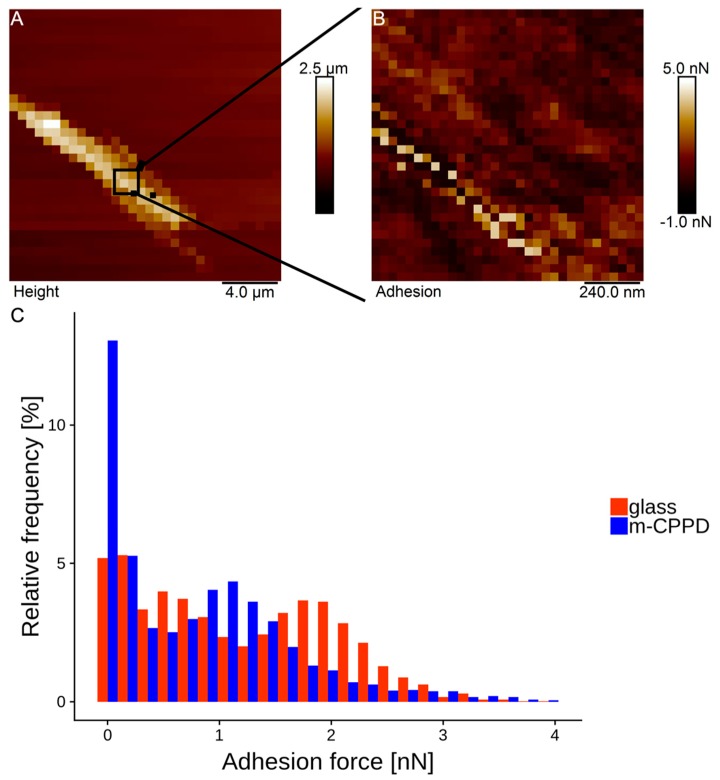
(**A**): AFM force volume height of m-CPPD crystal on epoxy, (**B**): adhesion map (64 × 64), (**C**): histogram of adhesion force maps (n = 3) for glass and m-CPPD crystals.

Atomic force microscopy (AFM) measurements have been performed to determine the interaction force between CPP and proteins and elucidate at the atomic level the adhesive properties of BSA protein on m-CPPD crystals. A method of AFM technique using chemical functionalization of AFM tips has been used to measure the adhesion forces between BSA and m-CPPD crystals. Preliminary tests highlighted the difficulty to obtain single crystals of m-CPPD. However preliminary results (Figure 7) show the sensitivity of the method that can distinguish between m-CPPD crystal and glass. m-CPPD highest adhesion peak is at 1.2 nN while that of glass is at 1.9 nN. This might indicate that BSA has much less affinity to m-CPPD than to glass for which it is known to have a good affinity. These results are in agreement with the parameters of the Langmuir-Freundlich model we determined, indicating a small quantity of adsorbed BSA and a low affinity of BSA for m-CPPD crystal surface.

## 3. Discussion

In this study we confirmed that protein adsorption altered the inflammatory properties of m-CPPD crystals. We showed that protein coating on MSU and m-CPPD crystals decreased the potential of these crystals to induce IL-1β production, the main cytokine that drove crystal-induced inflammation, by macrophages which were the cells that initiated the crystal inflammation process [46]. Our results suggested that protein coating decreased MSU and m-CPPD crystal-induced IL-1β production through NLRP3 inflammasome inhibition. Indeed, we observed that crystal-induced ATP production and mitochondrial membrane depolarization which were two known NLRP3 activators were modulated by protein coating [47]. As previously reported, the effects of protein coating on crystal-induced cell responses might be secondary to modulations of crystal/cell interactions [13,30,31,32,33,48,49,50,51,52,53,54,55]. In favor of this hypothesis was that the inhibiting coating effects disappeared when cells were stimulated during 15 min with uncoated crystals. These modifications of crystal/cell membrane interactions might also disturb NLRP3 inflammasome activation as reported by Ng et al. [41]. These latter authors showed that MSU crystals directly interacted with cell membrane leading to cell-membrane cholesterol and lipid sorting and Syk kinase and NLRP3 activation [41]. Similarly, Hari et al. observed that MSU and silica crystal-induced NLRP3 activation was independent of crystal phagocytosis but relied on crystal/cell membrane interaction leading to potassium efflux [40]. The interactions between crystals and cell membranes involved several mechanisms including electrostatic bindings between the negatively charged (zeta potential) crystal surfaces and cell-surface components, hydrogen or ion bindings and protein adsorption on crystal surfaces [13,19,21,22,23,24,25,26,27,28,29,32,33,37,48,49,50,51,52,53,54]. Interestingly, several authors had shown, first, that the inflammatory potential of MSU and m-CPPD crystals varied according to proteins coated on the crystal surfaces, and second, that the coated-proteins changed during inflammation states [13,19,21,22,23,24,25,26,27,28,29,32,33,37,48,49,50,51,52,53,54]. Thus, the inflammatory potential of MSU crystals increased with IgG coating while it decreased with ApoE or LDL coating [25,26,27,28,29,37]. Similarly, inflammation-induced by CPPD crystals was increased with IgG, plasma, serum or heparin coating but decreased with HDL or LDL coating [13,32,33]. Our results were in agreement with these former data and added new insights on the inhibition effects of BSA and serum coating on MSU and m-CPPD crystal-induced cell activation. Moreover, we identified using proteomic technology more than 30 proteins coated on m-CPPD crystal surface, most of them have never been described such as hemoglobin, ApO, complement and coagulation factors. Altogether, these findings gave new insights into pathogenesis of crystal-related rheumatic diseases and subsequently new therapeutic approaches. Thus, as described with nanoparticle-protein corona, crystal-protein interactions could be considered as a biological entity which interacted with immune cells in a biological system, hence influencing cell responses, macrophage uptake and fate and inflammatory responses [31,55]. Understanding the biological identity of crystal-protein complex, how it forms, changes and evolves in different clinical conditions, tissue environment and factors and how it modulates the immune response is necessary and vital to improve crystal-related disease care.

As albumin was present in high amount on m-CPPD crystal surface, we performed a physico-chemical study of BSA adsorption as protein model on m-CPPD crystals to elucidate the mechanism of protein/CPPD interaction and biological investigation results. Few studies had focused on CPPD/proteins interactions from a physico-chemical point of view, to our knowledge only Winternitz studied the adsorption of IgG protein on m-CPPD synthetic crystals and described the isotherms obtained by Freundlich equation which has traditionally been used to describe heterogeneous binding [56]. In the present paper, the mechanism of adsorption of BSA onto m-CPPD surface was well described by a Langmuir-Freundlich isotherm based on low quantity of proteins adsorbed and weak protein/m-CPPD crystal interaction in agreement with the low affinity of CPPD and MSU crystals with albumin already reported in the literature and also with the low adhesion forces between BSA and a m-CPPD crystal we measured by AFM [21,22]. We showed that the affinity of BSA for m-CPPD crystals was low whereas BSA was one of the proteins with the highest mascot score we determined by proteomic analysis. This apparent paradoxical result might be related to the strong interaction between protein molecules each other’s that seemed to occur. Indeed, these interactions between BSA molecules adsorbed on each m-CPPD crystals could explain crystals agglomeration observed by microscopy after in vitro cell tests and adsorption test (Figure 6). Whether the agglomeration state of the m-CPPD crystals could contribute to regulate the inflammatory response needs further investigations.

Such physico-chemical approach had never been reported in the literature to study CPPD crystals-proteins interactions. This physico-chemical study of BSA adsorption on m-CPPD crystals surface gave interesting results which could be extended to the other type of calcium pyrophosphate dihydrate clinically relevant (t-CPPD) and the precursor phases of CPPD (a-CPP and m-CPPTβ). Now the model of adsorption have to be confirmed on these other types of hydrated calcium pyrophosphate crystals and the influence of several parameters such as experimental conditions like the pH, the size and the agglomeration state of the crystals or the nature of the proteins have to be considered; particularly BSA was used as model of albumin protein but such approach can applied to the use of human serum albumin or other proteins of interest as hemoglobin, fibrinogen, complement and coagulation factors identified by proteomic analysis.

In conclusion protein adsorption on m-CPPD crystal surface might interfere on crystal/cell membrane interactions and modulated positively or negatively crystal-induced inflammation. The model of adsorption isotherm we determined, Langmuir-Freundlich isotherm, is in agreement with such hypothesis. Low amount of BSA is adsorbed on m-CPPD crystals with a weak BSA/m-CPPD crystal interaction but strong interaction between protein molecules each other’s favoring crystal agglomeration that might lower the inflammatory properties of m-CPPD crystals. Further investigations of these interactions could help to understand why CPPD deposit has so many clinical features.

## 4. Materials and Methods

### 4.1. MSU and m-CPPD Crystals Synthesis and Characterization

MSU crystals were obtained by spontaneous precipitation of uric acid in NaOH solution (0.01 M) at 60 °C as described [18]. m-CPPD pure phase was synthesized and characterized using a published protocol and methods [57]. The as-prepared m-CPPD powder was characterized by X-ray diffraction (Inel Equinox 1000 diffractometer with Co Kα radiation, Artenay, France), FTIR spectroscopy (Thermo Nicolet 5700 Fourier-transform infrared spectrometer, transmission mode with m-CPPD powder in KBr pellet) and scanning electron microscopy (SEM, Leo 435 VP microscope, Zeiss, Oberkochen, Germany, m-CPPD powder silver-plated before observation). Chemical analysis of pyrophosphate and calcium was performed as follows: standard spectrophotometric (Shimadzu UV1800 spectrometer, λ = 460 nm, Canby, OR, USA) determination of the yellow phosphovanadomolybdic acid complex was used to determine phosphate concentration after hydrolysis of the pyrophosphate (at 100 °C in acidic medium during 1 h) into phosphate ions; calcium concentration was determined by complexometry with ethylenediaminetetraacetic acid (EDTA). The specific surface area of the m-CPPD was evaluated using the Brunauer–Emmett–Teller method (nitrogen adsorption on a Monosorb Nova 1000, Quantachrome Instruments, Boynton Beach, FL, USA). This thorough physicochemical characterization of the synthetic calcium pyrophosphate dihydrate powder showed that it consists of a pure m-CPPD crystals (Figure A1 and Figure A2) [57].

Crystals/particles were dispersed by brief sonication and suspended at 2 mg/mL in phosphate buffered saline (PBS). They were prepared under endotoxin-free conditions and tested negative with Pierce Limulus amebocyte Assay (Thermo Fisher Scientific).

### 4.2. Mice

Adult Sv129 mice were used for in vivo experiments. Wild-type (wt) mice were purchased from Janvier Lab (Le Genest-St-Isle, France). Mice were maintained in cages (max. 6/cage) in a facility with 12 h light/dark cycles. Mice were fed diets ad libitum. All experiments were approved by the national ethical committee (#65352016110314184795V3).

### 4.3. Cells Culture

For in vitro experiments, bone marrow cells were recovered from tibia and femoral bones of mice and seeded in 24-well plates at 2 × 10^6^ cells/mL in L929-conditioned RPMI1640 media as described [43]. Every 2 days, cells were washed and the media renewed until complete differentiation of BMDMs. BMDMs were maintained in RPMI1640 supplemented with 10% fetal bovine serum (FBS), HEPES (25 mM), L-glutamine (2 mM), penicillin (100 U/mL), and streptomycin (100 µg/mL) and were primed overnight with ultrapure lipopolysaccharide (LPS; 20 ng/mL, Invivogene, San Diego, CA, USA).

Human monocytic leukemia (THP-1) cells were maintained in the same complete RPMI1640 media and were primed for 6 h with phorbol 12-myristate 13-acetate (PMA, 0.5 µM, Sigma-Aldrich, Saint-Louis, MO, USA), washed with PBS1X, then plated in 24-well dishes at 3 × 10^5^ cells/wells and left overnight in complete media.

Primed BMDMs and THP-1 cells were washed twice with PBS and stimulated at the indicated times with the synthetic MSU or m-CPPD crystals (200 µg/mL) in FBS-free media. For some experiments, crystals were incubated with FBS, 35% bovine serum albumin (BSA, Sigma Aldrich, Saint-Louis, MO, USA), mouse serum or blood lysate 30 min before stimulation then washed twice in PBS and resuspended at 2 mg/mL. After a 6 h (2 h for ATP dosage) crystal stimulation, supernatant was collected for cytokine quantification and cells were lyzed for mRNA quantification. Mouse blood was obtained by cardiac puncture, then blood was centrifuged 5 min at 4000 rpm at 4 °C, the supernatant was collected as serum and the pellet was lyzed to obtain blood cell lysate.

### 4.4. Cytokines and ATP Quantification

Cytokines and ATP production in supernatants were measured respectively by using IL-1β ELISA kits (Invitrogen, Carlsbad, California, USA) and ATP determination kit (Molecular probes, Eugene, OR, USA).

### 4.5. mRNA Quantification

Primed THP-1 cells were lyzed with TRizol reagent (Invitrogen), 6 h after crystal stimulation, and total RNA was extracted by using the ISOLATE II RNA kit (Bioline, London, UK). First, 500 ng of total RNA were reverse transcripted to cDNA using the High Capacity cDNA Reverse Transcription Kit (Applied Biosystem, Foster City, California, USA) (LifeECO Thermal Cycler, Bioer Technology, Hangzhou, Binjiang, China). Then, quantitative PCR was performed with 25 ng of cDNA using the SensiFAST SYBR No-ROX Kit (Bioline, London, UK) for 40 cycles (95 °C for 5 s, 60 °C for 30 s) (LightCycler®480 Instrument, Roche Life Science, Penzberg, Germany). Sequences of primers for qPCR are reported in Table 2.

### 4.6. Flow Cytometry

THP1 cells were plated in 24-wells dishes at 3 × 10^5^ cells/well, washed twice in PBS and stimulated with the synthetic MSU or m-CPPD crystals (200 µg/mL) in FBS-free media for 30 min then washed twice in PBS and incubated for 30 min in JC-1 (Molecular probes) then cells were washed twice in PBS and analyzed with the BD FACS Canto II cytometer (BD Bioscience, Franklin Lakes, NJ, USA). Data were analyzed with BDFACS Diva software (BD Bioscience, Franklin Lakes, NJ, USA).

### 4.7. Proteomic Analysis

m-CPPD crystals were incubated for 30 min in FBS at 37 °C then washed twice with FBS and finally crystals were collected by centrifugation. Proteins adsorbed on microcrystals were processed for mass spectrometry analysis without specific elution step. They were reduced with 10 mM DTT, alkylated with 55 mM iodoacetamide (IAA) and incubated with 20 μL of 25 mM NH_4_HCO_3_ containing 12.5 µg/mL sequencing-grade trypsin (Promega, Madison, WI, USA) overnight at 37 °C. Digests were pooled according to each samples and analyzed by a LTQ Velos Orbitrap (Thermo Fisher Scientific, San Jose, CA, USA) coupled to an Easy nano-LC Proxeon 1000 system (Thermo Fisher Scientific, San Jose, CA, USA). Chromatographic separation of peptides was performed with the following parameters: Acclaim Pepmap100 pre-column (5 mm, 300 μmi.d., C18, 5 μm, 100 Å) and column Easy Column Proxeon C18 (50 cm, 75 µm i.d., 120 Å), 300 nL/min flow, gradient rising from 95% solvent A (water, 0.1% formic acid) to 35% B (100% acetonitrile, 0.1% formic acid) in 97 min, then to 80% B in 6 min for a total run time of 118 min. Peptides were analyzed in the Orbitrap cell in full ion scan mode at a resolution of 30,000 (at m/z 400) and a mass range of 400–1800 m/z. Fragments were obtained with a collision-induced dissociation (CID) activation with a collisional energy of 40%, an activation Q of 0.25 for 10 ms, and analyzed in the LTQ. MS/MS data were acquired in a data dependent mode in which 20 most intense precursor ions were isolated, with a dynamic exclusion of 20 seconds and an exclusion mass width of 10 ppm.

For the protein identification step, all MS and MS/MS data were processed with the Mascot search engine (Matrix Science, version 2.5.1, London, UK). The mass tolerance was set to 7 ppm for precursor ions and 0.5 Da for fragments. The following modifications were used in variable parameters: oxidation (M), phosphorylation (Ser, Thr, Tyr), carbamidomethylation (Cys), deamidation (Asn, Gln), acetylation (N-term, Lys). The maximum number of missed cleavages was limited to 2 for trypsin digestion. The UniProt *Bos taurus* protein database was used for the identification step. Peptides with a Mascot score above 15 were considered.

### 4.8. Statistical Analysis

Data are reported as mean ± SEM. After verification of Gaussian distribution and homogeneous variance of each group, multiple t test followed by false discovery rate (FDR) correction were used to compare experimental conditions. Otherwise, Kruskal-Wallis test with FDR correction was chosen. The significance level was set at *P* < 0.05. GraphPad Prism 7.0 (San Diego, CA, USA) was used for analysis.

### 4.9. BSA Adsorption on m-CPPD Crystals

m-CPPD synthetic crystals (100 mg) have been dispersed in adsorption medium (5 mL), an aqueous isotonic solution (NaCl 0.15 M) of bovine serum albumin (BSA, Sigma Aldrich ref. A7030, Saint-Louis, MO, USA) at different concentrations (from 0 to 10 g/L, pH = 7.4, in triplicate). Blanks containing the adsorption solution without crystals were used as controls. The suspensions obtained have been sonicated (2 min) and incubated at 37 °C during 30 min without stirring to reach adsorption equilibrium, and then centrifuged (10 min, 5000 tr/min). The supernatants have been then filtered onto Millipore membrane (pore size 0.2 µm) and store before analysis. The solids (powders) have been washed and dried at 37 °C and then characterized.

Phosphorus and calcium contents in solution were determined using an inductively coupled plasma atomic emission spectrometer (ICP-OES, Thermo Electron, Iris Intrepid II XLD, Waltham, MA, USA), and BSA concentration by UV spectrophotometry (Shimadzu UV 1800 spectrometer, Canby, OR, USA) at 278 nm.

### 4.11. Preliminary Study of m-CPPD-BSA Interaction Force by Atomic Force Microscopy

Sample preparation: A drop of Epoxy glue (Loctite EA3430, Henkel Corp., Bridgewater, NJ, USA) was first spin coated (20 s at 800 rpm followed by 2 min at 6000 rpm) on a cleaned glass slide. The sample was then left to dry 50 min at 60 °C. A 200 µL drop of m-CPPD suspended crystals in de-ionized water (1.33 g/L) was then spin coated (20 s at 200 rpm followed by 30 s at 6000 rpm) on the epoxy glass slide and left to dry 15 min at 37 °C.

*AFM tip functionalization with BSA*: We used an AFM tip functionalization protocol reported by Jauvert et al. [58]. Briefly, this protocol makes use of dendrimer functionalized tips presenting CHO terminal functions that can react to link NH_2_ groups of proteins covalently. MLCT (Bruker, Karlsruhe, Germany) cantilever (nominal spring constant of 0.01 N/m) was used. The functionalized tip was then made to react with BSA proteins (Sigma Aldrich, Saint-Louis, MO, USA) prepared in solution (0.5 g/L in 0.1 M carbonate buffer at pH = 8.4).

*AFM data acquisition*: All measurements were performed in PBS 1X buffer solution (Sigma Aldrich, Saint-Louis, MO, USA) at 37 °C using a Bioscope Catalyst AFM (Bruker, Bruker, Karlsruhe, Germany). AFM cantilever sensitivity was calibrated on glass in PBS 1x buffer at 37 °C and its spring constant was determined using the thermal noise method [59]. First a rough (20 µm)^2^ 32 × 32 force volume mode image was performed on an isolated m-CPPD crystal (Figure 7A) allowing a more precise (1.2 µm)^2^ 128 × 128 force volume mode image on the top of the crystal to be acquired (Figure 7B). Data was acquired in triplicate on the top of three different crystals and on glass. The adhesion force data was extracted using Nanoscope Analysis 1.5 software (Bruker, Bruker, Karlsruhe, Germany) and plotted into a histogram (Figure 7C) using R open source statistical software.

## Figures and Tables

**Table 1:** List of the proteins with a mascot score higher than 40 identified on m-CPPD crystal surfaces after their incubation in FBS during 30 min at 37 °C.

Accession	Description	Σ Coverage	Mascot Score
62460494	hemoglobin fetal subunit beta [Bos taurus]	64.83	424.4
148745450	Fibrinogen alpha chain [Bos taurus]	32.85	312.2
116812902	hemoglobin subunit alpha [Bos taurus]	99.30	308.2
296474801	TPA: inter-alpha-trypsin inhibitor heavy chain H4 precursor [Bos taurus]	8.19	270.7
27806751	alpha-2-HS-glycoprotein precursor [Bos taurus]	21.73	225.2
74267962	ALB protein [Bos taurus]	16.14	185.5
1351907	Serum albumin Precursor	16.14	178.2
114052298	apolipoprotein A-II precursor [Bos taurus]	49.00	140.0
262050656	complement C4 precursor [Bos taurus]	3.96	138.4
75832056	apolipoprotein A-I preproprotein [Bos taurus]	58.11	137.5
268607679	coagulation factor XIII A chain [Bos taurus]	10.52	97.8
27819608	hemoglobin subunit beta [Bos taurus]	41.38	92.0
2501351	Serotransferrin Precursor	6.11	88.8
27806789	transthyretin precursor [Bos taurus]	40.82	82.8
47564119	apolipoprotein C-III precursor [Bos taurus]	44.79	82.0
51592135	cofilin-1 [Sus scrofa]	12.05	81.4
77735583	adenosylhomocysteinase [Bos taurus]	4.17	62.7
156139070	apolipoprotein C-II precursor [Bos taurus]	39.60	57.8
27806487	pigment epithelium-derived factor precursor [Bos taurus]	13.46	57.3
27807209	alpha-2-antiplasmin precursor [Bos taurus]	6.10	54.9
297488254	PREDICTED: serpin A3-3 [Bos taurus]	8.47	54.1
296487363	TPA: myosin, heavy chain 9, non-muscle [Bos taurus]	3.10	52.8
27807167	peroxiredoxin-6 [Bos taurus]	10.27	52.4
27807125	thymosin beta-10 [Bos taurus]	42.86	49.1
297466391	PREDICTED: alpha-2-macroglobulin, partial [Bos taurus]	1.90	47.2
296479148	TPA: alpha-enolase [Bos taurus]	13.82	43.5
114053019	alpha-1B-glycoprotein precursor [Bos taurus]	5.17	41.2
123959760	ubiquitin-like protein 4B [Bos taurus]	5.45	41.2
135806	Prothrombin Precursor	11.20	41.0

**Table 2:** List of primers.

	Forward	Reverse
IL-1β	TTCGAGGCACAAGGCACAA	TGGCTGCTTCAGACACTTGAG
IL-8	GAGCCAGGAAGAAACCACCG	TGGCAAAACTGCACCTTCACA
TNF-α	CCCATGTTGTAGCAAACCCTC	TATCTCTCAGCTCCACGCCA
Cox-2	GCTGTTCCCACCCATGTCAA	AAATTCCGGTGTTGAGCAGT
GAPDH	AGCCACATCGCCAGACAC	GCCCAATACGACCAAATCC
